# A case of collagenous gastritis and literature review: A case report

**DOI:** 10.1097/MD.0000000000033868

**Published:** 2023-05-26

**Authors:** Channi Wu, Lin Wang, Lan Zhang, Yangdan Ni, Zhiyong Wang, Yichen Qu

**Affiliations:** a Department of Gastroenterology, Affiliated Xiaoshan Hospital, Hangzhou Normal University, Hangzhou, China; b Department of Pathology, Affiliated Xiaoshan Hospital, Hangzhou Normal University, Hangzhou, China.

**Keywords:** collagenous gastritis, gastroscopy, hypoferric anemia, Masson staining

## Abstract

Collagenous gastritis (CG) is rarely encountered in clinical practice. Here, we reported a case of CG with iron-deficiency anemia as the main symptom.

**Patient concerns:**

The patient was a 26-year-old woman who sought medical advice with a chief complaint of recurrent upper abdominal distention and anemia since the last 3 years.

**Diagnoses:**

Gastroscopy at admission showed diffuse nodular mucosa. The pathology showed the formation of a belt hyperplasia of collagen in the superficial mucosa along with the infiltration of inflammatory cells. The subepithelial collagen band was 17.68 to 35.73-μm thick and tested positive for Masson staining, thereby confirming the diagnosis of CG.

**Interventions:**

A polysaccharide iron complex capsule was given in a dosage of 0.3 t.i.d., p.o. in combination with an omeprazole capsule (20 mg q.d. p.o).

**Outcomes:**

The symptoms (upper abdominal distention and anemia) were ameliorated after 8-week treatment. Blood routine showed that the hemoglobin level rose to 91 g/L.

**Lessons:**

It is difficult to diagnose CG. Hence, a comprehensive examination based on clinical manifestations, endoscopic findings, and pathological features is required.

## 1. Introduction

Collagenous gastritis (CG) is a rare disease. It was first reported in 1989 by Colletti and Trainer.^[1]^ Its pathology is characterized by the thickening of the subepithelial collagen band to more than 10 μm along with the infiltration by inflammatory cells.^[2]^ However, pathogenesis of this disease remains unclear. In addition, no definitive treatment for it has yet been developed. We herein report a case of CG with anemia as the main symptom. We also discuss related progress discussed in previous studies.

## 2. Case report

The patient, a 26-year-old woman, was presented to us on June 14, 2022, with the chief complaint of recurrent upper abdominal distention and anemia since the last 3 years. A physical examination showed an anemic appearance. The stomach seemed flat and soft to touch. The liver and spleen were not palpable. The patient denied any history of allergy. An adjuvant examination was conducted, which included a blood routine with the following results: white blood cells, 4.63 × 10^9^/L; hemoglobin, 86 g/L; hematocrit, 0.26 L/L; mean corpuscular volume, 77.7 fl; mean corpuscular hemoglobin, 25.5 pg; mean corpuscular hemoglobin concentration, 329.0 g/L; platelet 203 × 10^9^/L; and ferritin 2.6 ng/mL. Liver and kidney functions, tumor markers, coagulation function, thyroid function, IgG_4_, and autoantibodies were normal. No abnormality was noted in enhanced CT scans for the whole abdomen.

## 3. Endoscopy and pathology

**Figure 1.**
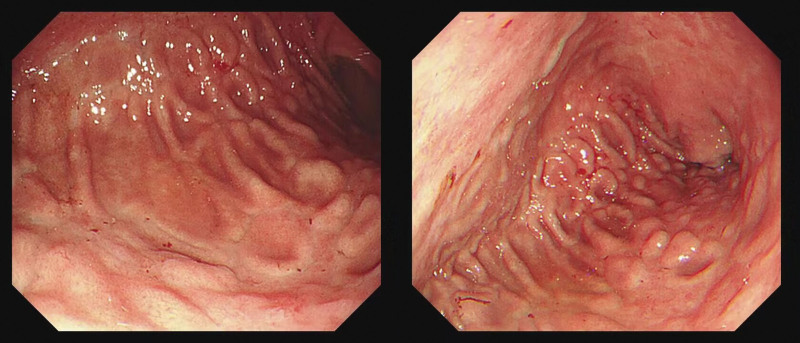
Gastroscopy: multiple island residual mucosa with surrounding atrophic changes are observed in the greater curvature of corpus ventriculi, where localized nodular eminences are noticed.

*Gastroscopy*: Multiple island residual mucosa and diffuse nodular mucosa were observed in the corpus ventriculi. The mucosa around the nodules was concave and atrophic (Fig. 1).

**Figure 2.**
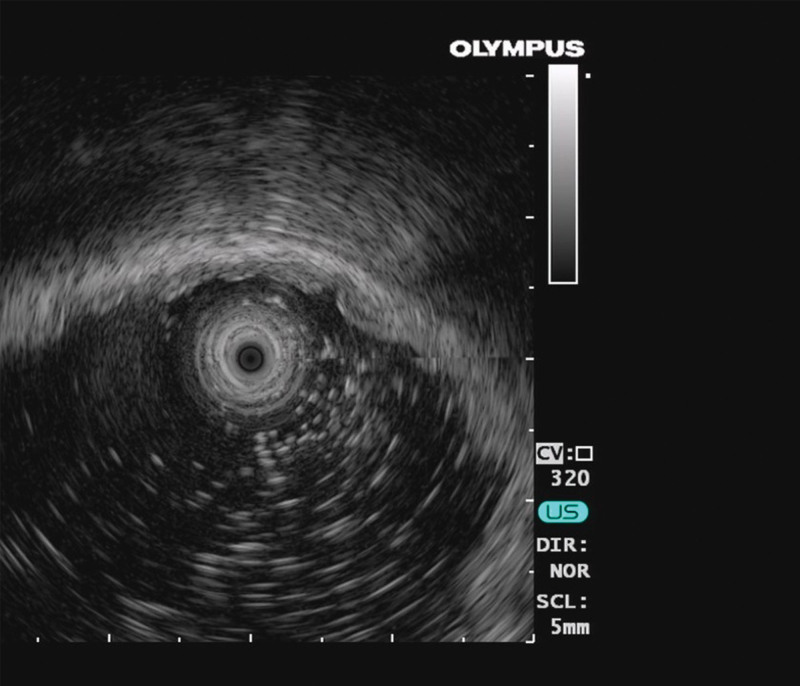
A clear structure of the gastric wall with a thickness of approximately 6.3 cm is observed using 12-MHz ultrasound. Both the mucosa and submucosa thickened, and the mucosa has a thickness of approximately 2.2 mm.

*Endoscopic ultrasonography*: Use of 12-MHz ultrasound afforded a clear structure with an approximately 6.3-mm-thick gastric wall. The thickness of the mucosa and submucosa increased. The mucosa was approximately 2.2-mm thick (Fig. 2). Colonoscopy did not show any obvious abnormality.

*Biopsy pathology*: The gastric mucosa showed atrophic changes, with atrophic and flat pits. The number of oxyntic glands in the corpus ventriculi also reduced.

**Figure 3.**
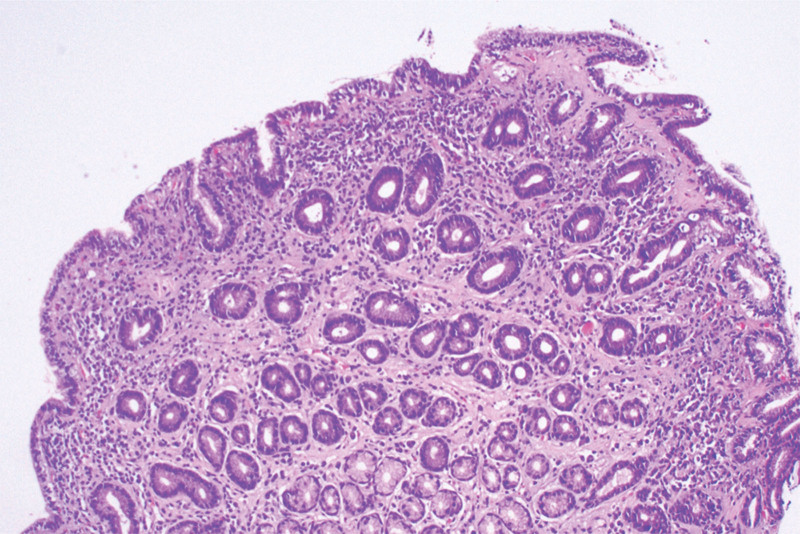
Mucosa shows atrophic changes, with a thickened collagen band appearing in the subepithelial layer. *Mesenchyma* is infiltrated with scattered lymphocytes, plasma cells, and eosinophils (×100; HE staining).

*HE staining*: The lamina propria was infiltrated with lymphocytes, plasma cells, and eosinophils (Fig. 3).

**Figure 4.**
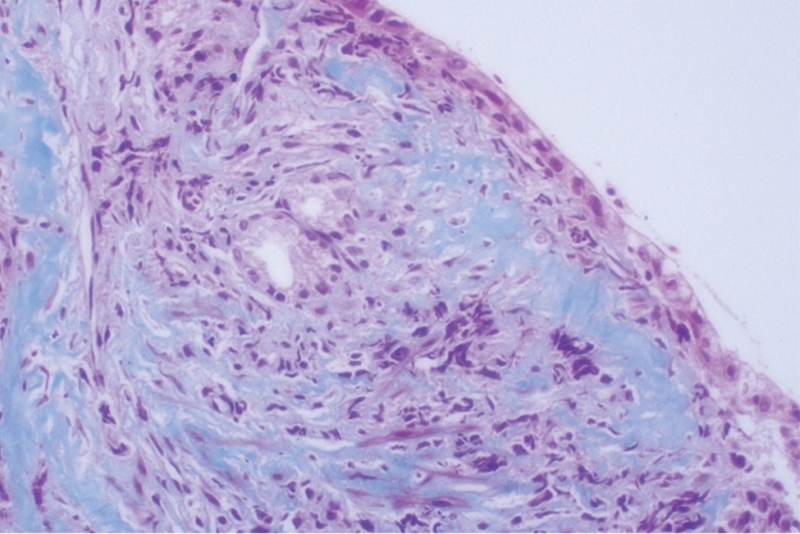
*Masson trichrome staining shows a* fibrotic band deposition of collagen in the subepithelial layer (in nattier blue) (×400).

*Masson staining*: The collagen band in the superficial mucosa showed a patchy distribution with a thickness of 17.68 to 35.73 μm (Fig. 4). Congo red staining was negative. The test for *Helicobacter pylori* was also negative. Pathological diagnosis was CG.

*Treatment and follow-up*: A polysaccharide iron complex capsule was given, in a dosage of 0.3 t.i.d., p.o. (manufactured by UCB [Zhuhai] Pharmaceutical Co., Ltd; Trade name: Niferex; [national medicine permission number: J20160027]) in combination with an omeprazole capsule (20 mg q.d. p.o. (manufactured by Zhejiang CONBA Pharmaceutical Co., Ltd]; Trade name: Kin-Kong [national medicine permission number: H20056062]). The symptoms (upper abdominal distention and anemia) were ameliorated after 8-week treatment. Blood routine showed that the hemoglobin level rose to 91 g/L.

## 4. Discussion

CG is a chronic persistent histological condition characterized through intermittent clinical courses.^[3]^ An epidemiological survey conducted in the United States reported the CG incidence to be approximately 13/100,000. The incidence was higher in women than it was in men. Its prevalence showed the profile of a bimodal age distribution, with its first peak occurring in young patients aged 10 to 19 years and the second appearing in women aged ≥ 60 years.^[4]^ Lagorce–Pages^[2]^ categorized CG into 2 sub-groups: children, in whom the disease manifests as severe anemia with lesions localized within the stomach and the nodular appearance of gastric mucosa, as observed via gastroscopy, and adults, in whom the condition is often complicated by *collagenous colitis. Chronic watery diarrhea has been identified as the main symptom.*

The characteristic endoscopic appearance of CG is nodular mucosa differing in size and distributed widely in the corpus ventriculi and the great curvature of the gastric antrum.^[5]^ It should be noted that in CG, it is not mucosa thickening but the sunken mucosa around the nodules that causes the typical nodular appearance through collagen deposition in concave mucosa and glandular atrophy induced by inflammation. The nodular lesions are the residual undamaged mucosa.^[6]^ The diagnosis of CG is mainly based on pathology results, which showed that the subepithelial layer is infiltrated with chronic inflammatory cells (including lymphocytes, plasma cells, and eosinophils), especially in the lamina propria, where the thickness of the collagen-band deposition exceeds 10 μm.^[6]^ Our case was a young woman with recurrent anemia and upper abdominal discomfort as the main symptoms. Gastroscopy showed diffuse nodular mucosa in the corpus ventriculi. Based on the result of biopsy pathology, the diagnosis of pediatric CG was confirmed.

The pathogenesis of CG is still unclear, perhaps owing to chronic inflammation, autoimmune reaction(s), abnormal proliferation of fibroblasts around the crypts, collagenization of exuded plasma protein,^[7]^ allergic reaction(s), drug-related reaction(s), and an increased number of IgG4 plasma cells.^[8]^ As shown by Käppi et al,^[9]^ about half of the first-degree relatives of these patients have autoimmune diseases and 40% of the patients produce autoantibodies. This finding supports the view of an autoimmune/immune-mediated mechanism in the pathogenesis of this disease. CG patients have hypoferric anemia, which is considered to be caused by the reduction of iron absorption caused by gastric-acid deficiency or other mechanisms.^[9]^ Therefore, the anemia symptoms of such patients can be ameliorated by iron supplementation.^[10]^ Consistent with these findings, the anemia in our patient also ameliorated after a treatment with chalybeate.

There is no well-established standardized therapeutic regimen for treating CG. Use of oral chalybeate combined with proton-pump inhibitors can improve the clinical symptoms in 78.5% of patients. Other treatments such as a gluten-free diet or corticosteroids also show a good clinical improvement rate, though histological relief is difficult to achieve.^[11]^ Fortunately, no malignant progress was found in the follow-up cases so far.^[12]^ These cases necessitate long-term follow-up and further investigation to clarify the pathogenesis of CG, which is conducive to formulate standardized therapeutic strategies.

## Acknowledgments

We thank Medjaden Inc. for scientific editing of this manuscript.

## Author contributions

**Formal analysis:** Lan Zhang, Yangdan Ni.

**Writing – original draft:** Channi Wu, Lin Wang, Yichen Qu.

**Writing – review & editing:** Channi Wu, Zhiyong Wang, Yichen Qu.
